# Radiosynthesis of no-carrier-added *meta*-[^124^I]iodobenzylguanidine for PET imaging of metastatic neuroblastoma

**DOI:** 10.1007/s10967-016-5073-1

**Published:** 2016-10-24

**Authors:** Michael Green, Jonathan Lowe, Manikandan Kadirvel, Adam McMahon, Nigel Westwood, Sue Chua, Gavin Brown

**Affiliations:** 1 Wolfson Molecular Imaging Centre, University of Manchester, Manchester, M20 3LJ UK; 2 Cancer Research UK, Angel Building, London, EC1V 4AD UK; 3 The Institute of Cancer Research, Sutton, London, Surrey SM2 5NG UK

**Keywords:** [^124^I], Radiochemistry, *m*IBG, *Meta*-iodobenzylguanidine, Positron emission tomography, Neuroblastoma

## Abstract

*Meta*-iodobenzylguanidine (*m*IBG) has been radiolabelled at the no-carrier-added level with [^124^I] for a proof of concept study to assess the diagnostic accuracy of [^124^I]*m*IBG PET/CT in detecting metastatic deposits in patients diagnosed with metastatic neuroblastoma. Radiolabelling of *m*IBG was achieved via the iododesilylation reaction between [^124^I]sodium iodide and *meta*-trimethylsilylbenzylguanidine. [^124^I]*m*IBG was produced in 62–70 % radioiodide incorporation yield from [^124^I]sodium iodide. The average amount of formulated [^124^I]*m*IBG was 359 MBq (range 344–389 MBq) with an average specific radioactivity of 4.1 TBq μmol^−1^ (range 1.8–5.9 TBq μmol^−1^) at end of synthesis.

## Introduction

Radioiodinated *meta*-iodobenzylguanidine (*m*IBG) is an analogue of the hormone and neurotransmitter norepinephrine. Its adrenergic uptake, storage and release properties allow its different radiolabelled forms to be clinically used for the radiotherapy and imaging of neuroendocrine tumours and cardiac diseases. Early studies using carrier-added [^123^I]*m*IBG with gamma scintigraphy to image normal myocardium function [1] have progressed to single photon emission computed tomography (SPECT) imaging of cardiac and pulmonary abnormalities using no-carrier-added [^123^I]*m*IBG [2, 3]. In comparison, limited diagnostic information can be obtained using planar scintigraphy with [^131^I]*m*IBG which is also used in radiotherapy to treat neuroendocrine tumours [4]. In contrast, positron emission tomography (PET) with [^124^I]*m*IBG [5, 6] is less widely used but has the potential to provide more detailed functional information concerning normal and diseased states of the neuroendocrine system.

Neuroblastoma is a neuroendocrine tumour which presents a significant health issue as the most common extracranial cancer in children. Its high risk form is associated with one of the worst prognoses of all childhood malignancies [6]. In vivo imaging for the accurate staging and measurement of response to therapy is critical to the risk stratification of patients with neuroblastoma in order to offer the most individually tailored treatment possible. Currently used planar [^123^I]*m*IBG scintigraphy does however have disadvantages associated with its poor spatial and anatomical resolution [7, 8]. SPECT has been used to provide better comparison with anatomical imaging data, in particular when images are fused with X-ray computed tomography (CT) or a combined SPECT-CT scanner is available. The disadvantages of SPECT are that resolution still remains relatively poor and quantification of uptake is difficult [9]. Additionally, rapid whole-body scanning is not possible with [^123^I]*m*IBG SPECT or SPECT/CT, imaging is time-consuming with a single body region taking around 30 min which is a particular problem in children [10]. Various combinations of X-ray computed tomography, [^99^Tc]bone scan, magnetic resonance imaging (MRI) and 2-deoxy-2-[^18^F]fluoro-d-glucose (FDG) with PET all provide valuable complementary scans however their use in combination is onerous for the patient and may involve several general anaesthetics.

**Scheme 1:**
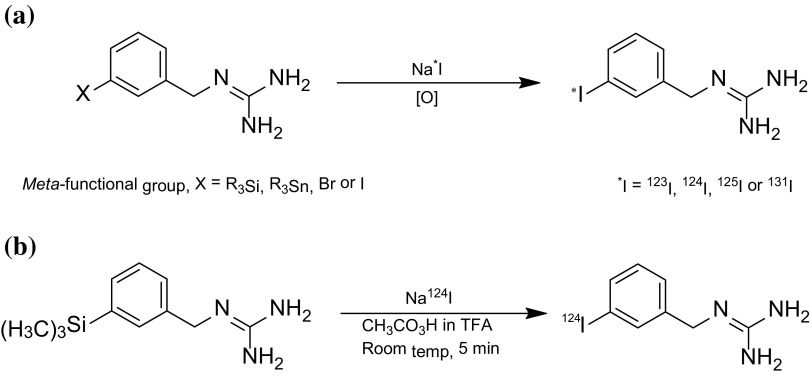
Synthesis of radioiodinated *meta*-iodobenzylguanidines

It has been proposed that [^124^I]*m*IBG PET/CT is more accurate than [^123^I]*m*IBG scintigraphy in detecting metastatic deposits in patients diagnosed with metastatic neuroblastoma. A proof-of-concept study can test this hypothesis by directly comparing the diagnostic performance of [^124^I]*m*IBG PET/CT with that of [^123^I]mIBG scintigraphy in patients with metastatic neuroblastoma. While commercial sources of [^123^I]*m*IBG are readily available, [^124^I]*m*IBG is produced only in a few radiochemistry facilities and would need to be prepared on demand for such a study. The first reported synthesis of [^124^I]*m*IBG describes a carrier-added process involving nucleophilic isotopic exchange on a solid phase support [11]. Subsequent advances in radiochemistry have led to several no-carrier-added methods to radioiodinated forms of *m*IBG via iododemetallation or iododebromination reactions (Scheme 1a) [12–15]. However only some of these methods have been applied to the radiosynthesis of [^124^I]*m*IBG. Notably, only low to moderate radiochemical yields of 15 and 44 % have been reported via [^124^I]iodination of a *tert*-butyloxycarbonyl (Boc) protected tin precursor [16]. This reaction is rather indiscriminate and generates a mixture of [^124^I]iodinated products, making chromatographic purification of [^124^I]*m*IBG very difficult. Here we report a method for the radiosynthesis of [^124^I]*m*IBG via an iododesilylation reaction which gives a [^124^I]product in good radiochemical yield and suitable for use in clinical PET research.

## Materials and methods

### Materials

[[3-(Trimethylsilyl)phenyl]methyl] guanidine sulphate (*m*TMSBG) was purchased from ABX advanced biochemical compounds (Dresden, Germany). Sodium iodide, trifluoroacetic acid, potassium phosphate (monobasic, Ph Eur grade), sodium bicarbonate, phosphoric acid, peracetic acid (32 % v/v in acetic acid) and ethanol were purchased from Sigma Aldrich Ltd (Gillingham, UK). 0.9 % sodium chloride was purchased from B Braun (Sheffield, UK). ^124^I as [^124^I]sodium iodide (*t*_1/2_ = 4.18 days, *β* ^+^ = 25.6 %) was supplied by Perkin Elmer and produced by BV Cyclotron VU (Amsterdam, The Netherlands) by a ^124^Te(*p*,*n*)^124^I reaction on enriched tellurium (^124^Te). It was isolated by dry distillation followed by chemical trapping in 0.2 M sodium hydroxide (~1.1 GBq ml^−1^).

### Methods

Mass spectra were recorded at the School of Chemistry, University of Manchester using the Micromass PLATFORM II (ES) and Thermo Finnigan MAT95XP (Accurate mass) instruments. Purification of products by semi-preparative HPLC was performed using a Shimadzu Prominence HPLC system controlled by Laura 3.0 software from LabLogic (Sheffield, UK). The system was configured with a CBM-20A controller, LC-20AB solvent delivery system, SPD-20A absorbance detector, FCV-20AH2 switching valve and Rheodyne^®^ 7725 injector. Radioactivity eluted by the HPLC system was monitored using a radio-HPLC Bioscan Flow Count B-FC 3100 detector. Radio-HPLC for quality control analysis was performed using a Shimadzu Prominence HPLC system configured with a CBM-20A controller, LC-20AB solvent delivery system, SPD-20A absorbance detector, SIL-20AHT autosampler and Bioscan Flow Count B-FC 3100 detector. Radioactivity measurements were made using an Isomed 2010 Dose Calibrator (MED Nuklear-Medizintechnik, Dresden, Germany).

### Synthesis of *m*IBG

A 300 μL sample of 150 μM sodium iodide in 0.2 M sodium hydroxide was evaporated to dryness in a V-shaped Wheaton Vial (2.0 mL) under a stream of helium gas at 100 °C. (total drying time = 20 min). The dried residue was cooled to 25 °C followed by the addition of a solution of *m*TMSBG (0.25 mg, 0.92 μmol) in trifluoroacetic acid (250 μL). A 20 μL aliquot of 32 % v/v peracetic acid in acetic acid was then added and the mixture was stirred at 25 °C for 5 min. The resultant mixture was then diluted with 1.5 mL of 0.6 M potassium phosphate (monobasic) and injected onto an HPLC column (Phenomenex Bondclone C_18_, 250 × 4.6 mm i.d.). The column was eluted at a flowrate of 2.0 mL min^−1^ using a mixture of 0.1 % phosphoric acid:ethanol (85:15 v/v). The mobile phase was monitored continuously for both radioactivity and UV absorbance at 254 nm. The fraction eluting at 14 min, having the same retention time as reference *m*IBG, was collected.

### Characterisation and analysis of *m*IBG

Samples of *m*IBG collected after HPLC purification were analysed by HPLC using an Ace 3 Phenyl column, 3 μm (150 × 4.6 mm i.d.) eluted at 1.0 mL with a mixture of 50 mM sodium dihydrogen phosphate solution, pH 7.0 and methanol (52.5:47.5, v/v). The eluate was monitored continuously for UV absorbance at 205 nm. The *m*TMSBG precursor eluted at 11.50 min and benzylguanidine eluted at 3.25 min. A single product peak with the same retention time (5.00 min) as authentic *m*IBG was observed. Mass spectrometry of the product (ES+ve mode) gave a peak at *m*/*z* = 276 [M+H]^+^.

### Synthesis of [^124^I]*m*IBG

Typically, a 200–300 μL sample of [^124^I]sodium iodide (555–600 MBq at the start of the radiosynthesis) in 0.2 M sodium hydroxide was evaporated to dryness in a V-shaped Wheaton Vial (2.0 mL) under a stream of helium gas at 100 °C. (drying time = 20 min). The dried residue was cooled to 25 °C followed by the addition of a solution of *m*TMSBG (0.25 mg, 0.92 μmol) in trifluoroacetic acid (250 μL). A 20 μL aliquot of 32 % v/v peracetic acid in acetic acid was then added and the mixture was stirred at 25 °C for 5 min. The resultant mixture was then diluted with 1.5 mL of 0.6 M potassium phosphate (monobasic) and injected onto an HPLC column (Phenomenex Bondclone C18, 250 × 4.6 mm i.d.). The column was eluted at a flowrate of 2.0 mL min^−1^ using a mixture of 0.1 % phosphoric acid:ethanol (85:15 v/v). The mobile phase was monitored continuously for both radioactivity and UV absorbance at 254 nm. The fraction eluting at 14 min, having the same retention time as reference *m*IBG was collected into a vial containing 16 mL of 0.9 % saline and 100 μL of 8.4 % sodium bicarbonate. Under aseptic conditions the resultant solution was then dispensed 3 ways via a Millipore filter (0.22 µm, Millex GS, Millipore) into sterile vials as 2.0 and 6.0 mL samples for quality control analysis and a 10.0 mL sample for PET imaging application. The total radiosynthesis time was 75 min.

### Quality control analysis of [^124^I]*m*IBG

Samples of [^124^I]*m*IBG formulated in normal saline for human injection were analysed by HPLC using an Ace 3 Phenyl column, 3 μm (150 × 4.6 mm i.d.) eluted at 1.0 mL min^−1^ with a mixture of 50 nM sodium dihydrogen phosphate, pH 7.0 and methanol [52.5:47.5, v/v]. The eluate was monitored continuously for radioactivity and for absorbance at 205 nm. The retention time for the *m*TMSBG precursor was 11.50 min. A single radioactive peak with the same retention time (5.00 min) as authentic *m*IBG was observed. Radiochemical and chemical purity of the formulated product was greater than 99 %.The bacterial endotoxin content of the product solution was measured using an Endosafe portable test system (Charles River, L’Abresle, France). Gas chromatography analysis of residual solvents (ethanol, acetone) was performed using a Shimadzu GC-2010 gas chromatograph fitted with an Rtx-624, 30 m, 0.32 mm, 1.8 μm column (Thames Restek, High Wycombe, UK) and a flame ionisation detector. The injection (2.5 μL sample) port temperature was 220 °C and the column flow rate was 1.4 mL min^−1^. Samples were also tested for pH and visually inspected to assess their colour and clarity. For product stability studies the quality control analysis of three consecutive batch samples was repeated at 0, 24, 48, 72, 96, 120, 144 and 168 h from the end of the radiosynthesis. In between each analysis the samples were stored under sealed conditions at 25 °C.

## Results and discussion

This work was undertaken to support an ongoing multicentre Phase I/II clinical trial to compare the diagnostic performance of [^124^I]*m*IBG PET/CT with that of [^123^I]*m*IBG scintigraphy in patients with metastatic neuroblastoma. The trial requires that samples of [^124^I]*m*IBG be prepared on demand and transported long distances followed by overnight storage prior to use. It was therefore necessary to have a reliable radiosynthesis method which produces [^124^I]*m*IBG with high radiochemical stability and room temperature chemical stability beyond 24 h after radiosynthesis.

There are a number of possible synthesis methods to [^124^I]*m*IBG however some involve carrier-added processes generating excessive amounts of stable product [17]. Other methods use a resin bound starting precursor [18] which cannot be purchased commercially and might prove too difficult to prepare. Of the methods surveyed, the high yielding iododesilylation reaction [12] between sodium iodide and *meta*-trimethylsilylbenzylguanidine (*m*TMSBG) was selected as the most favourable route. However, one concern was that the acidic reaction conditions required might give rise to precursor decomposition via loss of the trimethylsilyl group or other side reactions. A sub-micromolar scale method was therefore developed to create reaction conditions which could be translated to [^124^I] radiochemistry and also test for stable decomposition products. Under dried conditions, sodium iodide was reacted with *mTMSBG* at 25 °C. Analytical scale HPLC was then used to isolate the product in solution. Under the HPLC conditions used the *m*TMSBG precursor was strongly retained on the HPLC column and eluted at 27 min. No benzylguanidine (retention time = 7 min) was detected as might be expected if desilylation of the *m*TMSBG precursor had occurred. *M*IBG eluted as the sole product at 14 min. The eluent fraction containing the product was evaporated to dryness, redissolved in ethanol and analysed by mass spectrometry (ES+ve mode) and gave a single peak at *m*/*z* = 276 [M+H]^+^ (*m*IBG: C_8_H_10_IN_3_ = 275 formula weight).

**Fig. 1:**
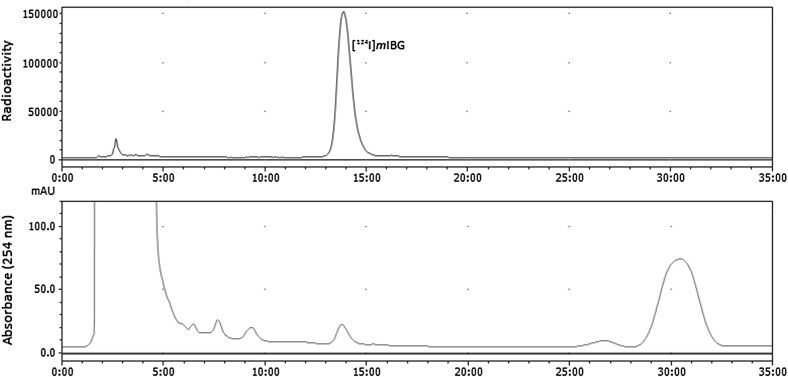
A typical HPLC chromatogram from the radiosynthesis of [^124^I]*m*IBG

The same sub-micromolar scale method conditions used to make mIBG were then applied to the synthesis of [^124^I]*m*IBG using commercially sourced [^124^I]sodium iodide. Using this method *m*IBG was labelled in the *meta* position with [^124^I] via the iododesilylation reaction between [^124^I]sodium iodide and *meta*-trimethylsilylbenzylguanidine (*m*TMSBG) (Scheme 1b). The radio-HPLC profile of the reaction mixture shows [^124^I]*m*IBG as the sole radioactive product and unreacted [^124^I]sodium iodide (2.50 min) as the only other radioactive species (Fig. 1). [^124^I]*m*IBG was produced typically in 62–70 % radioiodide incorporation yield from [^124^I]sodium iodide. By comparison it should be noted that the radioiodide incorporation yield for corresponding [^131^I] reaction is 90 % [12]. However similar differences in radioiodination yields due to a change in radioisotope have previously been reported. For example, the radioiodination of *N*-hydroxysuccinimidyl-4-tributylstannylbenzoate proceeds in 60–70 % yield when labelled with [^125^I] compared to 20–40 % with [^124^I] [19]. However in this case the [^124^I]iodination yield remains high for the synthesis of [^124^I]*m*IBG and generates ample amount of [^124^I]product for PET application. In summary, the average amount of [^124^I]*m*IBG formulated for clinical use was 359 MBq (range 344–389 MBq) with an average specific radioactivity of 4.1 TBq μmol^−1^ (range 1.8–5.9 TBq μmol^−1^) at EOS (end of synthesis) corresponding to on average 24 ng; 88 pmol (range 18–53 ng; 66–191 pmol) of carrier *m*IBG (n = 11).

A stability study of the formulated [^124^I]*m*IBG was performed over 7 days to test its suitability for transportation, overnight storage and to define a shelf life and storage temperature. Three formulated samples of [^124^I]*m*IBG were prepared and tested for pH, solution colour, solution clarity, endotoxin content, residual solvents, chemical purity and radiochemical purity. Each sample was tested at the end of synthesis then retested at 24 h intervals over 7 days. In between testing the samples were stored under sealed conditions at 25 °C. At all of the test time points all three samples conformed to agreed product specification criteria (Table 1) however all three samples underwent a small amount of radiolysis. Thus it was observed that all three samples maintained a radiochemical purity of 95 % or greater for up to 48 h after radiosynthesis (Table 2). After 168 h the radiochemical purity of two of the samples had fallen to 93 % or lower and one sample had a radiochemical purity of 96 %. The results also indicate that the rate of radiolysis is directly proportional to the amount of radioactivity in each 10 mL sample at the end of synthesis. Free [^124^I]iodide was detected as the only radiochemical impurity in all of the formulated samples tested. Based on the results obtained a shelf life of 96 h from the end of synthesis was set when the product is stored at ≤25 °C.

**Table 1:** Quality control acceptance criteria for [^124^I]*m*IBG

Test	Method	Acceptance criteria
Identity	Radio-HPLC	Conforms with authentic *m*IBG reference
Appearance	Visual inspection	Clear and colourless
pH	pH paper test	5–8
Radionuclidic purity	Gamma spectroscopy	[^124^I] accounts for >99.9 % of total radioactivity
Radioactivity concentration at the end of synthesis	Dose calibrator	8.4–18.5 MBq mL^−1^
[^124^I]*m*IBG radiochemical purity	Radio-HPLC	>90 %
mIBG content	Radio-HPLC	≤5.0 μg mL^−1^
*m*TMSBG content (+ unknown impurities)	Radio-HPLC	≤1.0 μg mL^−1^ of *m*TMSBG ≤1.0 μg mL^−1^ of unknown impurities (measured relative to *m*IBG)
Residual solvents	GC	
Bacterial endotoxins	Limulus amebocyte lysate (LAL) test	≤1.0 EU kg^−1^ mL^−1^
Filter integrity testing	Bubble point test	≥50 psi minimum bubble point pressure

**Table 2:** Radiochemical stability data for [^124^I]*m*IBG

Sample	[^124^I]*m*IBG radioactivity at the end of synthesis in 10 mL sample volume (MBq)	Radiochemical purity (%)							
		0 h	24 h	48 h	72 h	96 h	120 h	144 h	168 h
1	63.9	100	100	100	100	100	100	96	96
2	130.6	100	100	100	96	96	95	95	93
3	191.9	100	96	95	93	93	90	<90	<90

## Conclusions

*Meta*-iodobenzylguanidine was labelled in the *meta* position with [^124^I] via the radical catalysed iododesilylation reaction between [^124^I]sodium iodide and *meta*-trimethylsilylbenzylguanidine. Stability tests show that the formulated [^124^I]product is of high radiochemical purity (≥95 %), undergoes minimal radiolysis (<5 %) and is safe to inject into humans up to 48 h after production.
